# An Examination of Risk Factors for Tobacco and Cannabis Smoke Exposure in Adolescents Using an Epigenetic Biomarker

**DOI:** 10.3389/fpsyt.2021.688384

**Published:** 2021-08-24

**Authors:** Allan Andersen, Meg Gerrard, Frederick X. Gibbons, Steven R. H. Beach, Robert Philibert

**Affiliations:** ^1^Department of Psychiatry, University of Iowa, Iowa City, IA, United States; ^2^Department of Psychological Sciences, University of Connecticut, Storrs, CT, United States; ^3^Center for Family Research, University of Georgia, Athens, GA, United States; ^4^Department of Psychological Sciences, University of Georgia, Athens, GA, United States; ^5^Behavioral Diagnostics, Coralville, IA, United States

**Keywords:** smoking, epigenetics, DNA methylation, AHRR, cg05575921, digital PCR, adolescent

## Abstract

**Objective:** Evolving patterns of nicotine and cannabis use by adolescents require new tools to understand the changing epidemiology of these substances. Here we describe the use of a novel epigenetic biomarker sensitive to both tobacco and cannabis smoke in a longitudinal sample of high-risk adolescents. We examine risk factors for positivity for this epigenetic biomarker in comparison to positivity for conventional serum biomarkers of nicotine and cannabis use.

**Method:** Eastern Iowa 10th graders who had a friend or family member who smoked were eligible to participate in a longitudinal study over 10–12th grades. Subjects provided self-report data on nicotine, tobacco, and cannabis use patterns as well as blood samples that were used for serum cotinine and THC assays. DNA was prepared for analysis of methylation at the CpG cg05575921, a sensitive indicator of smoke exposure. Relationships between positivity for each these biomarkers and a variety of risk factors, including demographics, family and peer relationships, psychopathology, willingness to smoke, and perceptions of typical cigarette and cannabis users, were examined at the 10th (*n* = 442), 11th (*n* = 376), and 12th (*n* = 366) grade timepoints.

**Results:** A increasing proportion of subjects were positive for cotinine (5–16%), THC (3–10%), and cg05575921 methylation (5–7%) across timepoints, with some overlap. Self-reported combusted tobacco and cannabis use was strongly correlated with all biomarkers, whereas cg05575921 methylation was not correlated with reported e-cigarette use. Dual users, defined as those positive for nicotine and THC in the 12th grade showed the greatest cumulative smoke exposure, indicated by cg05575921 methylation. Subjects reported more positive attitudes toward cannabis users than cigarette smokers, and willingness to smoke and positive perceptions of tobacco and cannabis smokers were significant risk factors for biomarker positivity across timepoints.

**Conclusion:** We conclude that measurement of cg05575921 methylation in adolescents is a useful tool in detecting tobacco smoking in adolescents, and may be a novel tool for the detection of cannabis smoking and cannabis and tobacco co-use, though non-combusted forms of nicotine use do not appear to be detectable by this method.

## Introduction

The landscape of adolescent smoking has seen significant changes over the past three decades. Since 1991, past 30-day cigarette use by 18-year-olds in the US has declined from 18.5% to under 5%, while the proportion disapproving of cigarette use increased from 70% to nearly 90%, and the proportion viewing smoking as having a “great” risk increased from 50 to 75% (1). Concurrently, while the proportion of 12th graders reporting regular cannabis use held steady at around 6%, the proportion disapproving of cannabis decreased from roughly 90 to 65%, and the proportion viewing regular cannabis use as risky decreased from nearly 80% to under 30%. Thus, while the decrease in adolescent smoking is rightly viewed as a public health success, increasingly positive perceptions of cannabis along with expanding legalization of cannabis use across the US (2) are concerning trends.

Tobacco and cannabis co-use, e.g., cannabis smoked in a tobacco cigar as a “blunt,” is a trend that has also prompted significant concern, as co-use is associated with worse overall outcomes than either alone (3, 4). While this form of use has been shown to be particularly prevalent among African-American youth (5, 6), it remains poorly defined and understudied (7). Some studies report that blunt smokers perceive them to be more “natural,” less addictive (8, 9), more socially acceptable (10, 11), and less risky than cigarettes (12). In fact, nicotine appears to heighten the reinforcing effects of cannabis (13), increasing the risk of subsequent addiction (14). Studies have also shown that cannabis users are more likely to go on to smoke cigarettes (15), while cigarette users are more likely to use cannabis (16).

Lastly, the rise of e-cigarette use by adolescents is an emerging public health crisis (17), with past 30-day use among 12 graders estimated at 25% by the Monitoring the Future study (18). E-cigarettes carry acute risks of lung injury in some users (19) and longer-term risks of progression to nicotine dependence and unintended transitioning to combusted tobacco use (20). E-cigarette use has also been linked to a higher risk of using vaporized cannabis products (21), which may in turn predispose to cannabis dependence and other risks.

In this rapidly evolving landscape, better tools are needed in order to monitor adolescent smoking patterns, both epidemiologically and clinically. Unfortunately, detecting adolescent substance use is not easy. The most commonly-used method, self-report, suffers from poor accuracy in adolescents, likely due to both stigma and illegality (22). For example, one study of 367 adolescents self-report of cannabis use status, determined by urinalysis, was only 64% sensitive (23), and another study reported that only two-thirds of youth with detectable serum cotinine admitted to smoking in the past 5 days (24). Reliability of self-report may be even lower in minorities (25), though in some cases self-reporting may be confounded by the fact that blunt smokers may not identify as “tobacco” users or be aware of their nicotine exposure (26).

Limitations of objective biomarkers of substance relate primarily to their short half-lives of detection (27). Exhaled carbon monoxide (CO), though easy to measure, is detectable for only 4–5 h and is insensitive to light smoking (28). Cotinine, the primary metabolite of nicotine, can be assayed in saliva, blood or urine, and has a longer half-life of 15–19 h but similarly lacks sensitivity in the sporadic smoking which is typical of adolescents (29); its metabolism may vary by ethnicity (30, 31). In addition, cotinine assays cannot distinguish the source of nicotine exposure, whether combusted cigarette, e-cigarette, or nicotine replacement therapy (NRT).

Recent advances in the field of epigenetics may offer a means of overcoming some limitations of current smoking biomarkers. In particular, multiple studies and meta-analyses (27, 32, 33) have shown that decreased methylation of the genomic CpG cg05575921, located in the gene *Aryl Hydrocarbon Receptor Repressor* (*AHRR*), is a strongly performing biomarker for cigarette (AUC = 0.99) (34) that is both sensitive to light smoking (35) and demonstrate dose-response characteristics (34, 36, 37).

Importantly, while demethylation of cg05575921 appears to be driven by exposure to polyaromatic hydrocarbons (PAHs) and other compounds of tobacco smoke, this mechanism does not appear to be specific to combusted tobacco alone (38–42). Both tobacco (43) and cannabis (44) smoke contain high levels of PAHs, which when inhaled increase expression of the *Aryl Hydrocarbon Receptor* (*AHR)*, and subsequently *CYP1A1* (45), facilitating detoxification of these compounds. Lastly, expression of *AHRR* increases as a regulatory response to increased AHR expression (40), which if unregulated may result in carcinogenesis (39).

In this study we explore the epidemiology of this novel epigenetic biomarker for smoke exposure in a longitudinal cohort of high-risk adolescents in Eastern Iowa. We examine a number of risk factors traditionally associated with tobacco and cannabis smoking including race (46) ethnicity (47), SES (48), parental education (48), parental supervision (3, 49), parental smoking (50), peer use (51, 52), significant other use (53), and both externalizing and internalizing psychopathology (54–57). We also examine two variables from the Prototype/Willingness Model of adolescent risk behavior that have been shown to predict non-intentional but volitional adolescent risk behaviors: perceptions of prototypical adolescent cigarette and cannabis smokers (58), and willingness, an acknowledgment that under certain circumstances, one might engage in a risk behavior that was previously not intended or sought (59). Lastly, we examine the relationships between the conventional biomarkers of tobacco and cannabis smoking, serum cotinine and THC, and cg05575921 methylation, an epigenetic biomarker of smoke exposure.

Because measurable demethylation of cg05575921 reflects cumulative rather than short-term smoke exposure, we hypothesize that the number of significant risk factors for epigenetic positivity would increase over time. In addition, we hypothesize that co-users of tobacco and cannabis will demonstrate the greatest cumulative exposure to toxicants, as indicated by greater change in cg05575921 methylation.

## Materials and Methods

### Subjects

Subjects were drawn from the Healthy Iowans Study, a longitudinal study of adolescents in Eastern Iowa focusing on nicotine use and related risk factors and risk behaviors. Study procedures and protocols were approved by the University of Iowa's Institutional Review Board (IRB ID # 201409705). To ensure confidentiality, a NIH Certificate of Confidentiality was obtained.

Recruitment procedures for the study have been previously described (60). In brief, subjects were sophomores attending one of seven Eastern Iowa high schools and were provided with information about the study through the school. Interested subjects were contacted by study staff who provided further information and completed enrollment along with their parent/guardian. A bilingual staff member was available for Spanish speaking participants or families. Prior to consent, each adolescent subject and their parent or guardian were informed that study procedures would include blood tests for the presence of nicotine and cannabinoid by-products, and that these would remain confidential and only be available to study staff in a de-identified form.

After consent, each subject was interviewed by a trained research assistant using an abbreviated child version of the Semi-Structured Assessment for the Genetics of Alcoholism (SSAGA) (61, 62). Subjects were asked about their use of cigarettes, e-cigarettes, and cannabis over their lifetime and in the past year. Subjects also reported on the presence or absence of DSM-5 symptoms of Attention-Deficit Hyperactivity Disorder, Oppositional Defiant Disorder, and Conduct Disorder. The Patient Health Questionnaire-9 (PHQ-9) was included as a measure of depressive symptomatology. Supplemental interview questions, based on the Prototype/Willingness Model of Adolescent Risk Behavior, asked subjects to rate their willingness to smoke one cigarette or one cannabis “joint,” and their perceptions of peers who smoke cigarettes and smoke cannabis (59). Of note, questions on adolescents' self-reported cannabis use were inadvertently omitted from the interview at the 11th grade timepoint and thus not available for analysis.

Following the administration of the study interview, phlebotomy was performed to provide DNA from peripheral blood cells and serum for ELISA-based analysis of substance exposure.

### Assay Procedures

DNA extraction from whole blood was performed according to our previously published protocols (60), then frozen at −80°C until usage (63).

To determine recent smoke exposure *via* measurement of methylation at cg05575921 (34), 1 μg aliquots of DNA were first bisulfite converted using an Epitect Fast 96 Bisulfite Conversion kit (Qiagen, Germany). Next, converted DNA samples were pre-amped, diluted 1:3000, and then PCR amplified using fluorescent, dual labeled primer probe sets specific for cg05575921 obtained from Behavioral Diagnostics (Coralville, IA, USA) through their distributor IBI Scientific (Dubuque, Iowa, www.ibisci.com) and Universal Digital PCR™ reagents and protocols were obtained from Bio-Rad (Carlsberg, CA, USA). PCR reactions were performed on a Bio-Rad droplet digital PCR (ddPCR), which allows for reference-free measurement of allele proportions (64). In the current study, the alleles of interest were the “C” alleles, corresponding to the methylated cg05575921 cytosine residue, and the “T” allele, corresponding to the unmethylated cg05575921 cytosine residue. The proportion of each allele was analyzed using Bio-Rad's proprietary QuantiSoft™ software and expressed as percent methylated for use in subsequent analyses.

Serum cotinine and cannabinoid values were determined by enzyme linked immunoassay (ELISA) using kits from AbNova (Taiwan) and read on Molecular Devices (Sunnydale, USA) EMax spectrophotometer. All samples were frozen at −80°C until usage (63).

### Follow-Up Visits

Repeat venipuncture to obtain whole blood for DNA preparation and serum samples for analysis of substance use were performed again at the 1 year (11th grade) and 2 year (12th grade) timepoints.

### Coding Procedures and Data Analysis

All data storage and analyses were conducted on password-protected computers using R version 4.0.0 (65). Epigenetic positivity for smoke exposure was defined as cg05575921 methylation below 80% methylation, a cutoff chosen based on our previously published work for its combination of high sensitivity and specificity (34). Cotinine positivity was defined as a serum value of ≥3 ng/mL, while THC positivity was defined as ≥0.5 ng/mL.

Subjects reported on environmental risk factors for smoking; their answers were subsequently dichotomized as “high” or “low” risk for use in subsequent analyses. Most questions included four possible responses, corresponding to numeric scores of 1–4 and in these cases, 1 and 2 were typically coded as “low” and 3 and 4 as “high” risk. Yes/no questions required no further dichotomization. Responses to whether a subject had a mother, father, or sibling in the home who smoked were dichotomized to “any” or “no” family members in the home who smoked.

Willingness questions asked subjects whether they would be “not at all willing,” “kind of willing,” or “very willing” to smoke a cigarette or a “joint” of cannabis. For analysis, these responses were dichotomized into “low” risk (“not at all” or “kind of” willing) vs. “high” risk (“very willing”).

Prototype questions asked subjects to rate peers who smoked cigarettes and used cannabis by how “popular,” “smart,” “cool,” “attractive,” and “dull or boring” (reversed) they were, with options including “not at all,” “a little bit,” “kind of,” and “very,” which corresponded to numeric scores of 1–4. These “Prototype Scores” were then totalled for each substance and the subjects scoring approximately in the top 25th percentile (cutoffs varied depending on the distribution of scores) were coded as being at “high” risk, while the remainder were coded as “low” risk.

The final set of risk factors examined included symptoms of Attention-Deficit Hyperactivity Disorder (ADHD), Oppositional Defiant Disorder (ODD), Conduct Disorder (CD), and Major Depressive Disorder (MDD) (66). Subjects were coded as having a “high” level of ADHD symptoms if they endorsed ≥6 symptoms in the inattentive and/or hyperactivity/impulsivity categories, a “high” level of ODD symptoms if they endorsed ≥4 symptoms, and a “high” level of CD symptoms if they endorsed ≥3 symptoms. Depressive symptoms were assessed via the PHQ-9 and coded as “high” if they scored ≥10 (67).

Following dichotomization of the above variables, 2x2 tables were constructed for each biomarker x risk factor at each of the three timepoints. Odds Ratios (ORs) were then calculated for each 2x2 table using the *odds ratio.wald()* function in the *epitools* package (68) in R.

Spearman correlations between select variables were calculated using the *cor.test()* function in R. Linear regression models predicting cg05575921 methylation and ANOVA comparisons between groups were calculated using base packages in R.

## Results

### Subject Characteristics

A total of *n* = 448 subjects enrolled in the study and completed interviews and biomaterial sampling. DNA methylation analyses of cg05575921 were completed for a total of *n* = 442 subjects at the 10th grade timepoint. Of these, 376 (85.1%) returned for the 11th grade assessment and successfully completed DNA methylation analysis, and 366 (82.8%) returned for final assessment in 12th grade and completed DNA methylation analysis. Of these, 437 were successfully assayed for cotinine and 440 for THC at the 10th grade visit; 377 were successfully assayed for serum cotinine and THC at the 11th grade visit, and 364 successfully assayed for serum cotinine and THC at the 12th grade visit.

Clinical and demographic characteristics of the subjects are shown in Table 1. At study intake in 10th grade, subjects' age was tightly distributed around 16 years, and the majority were female (55%). The majority were white (84%), and non-Hispanic (84%). Subjects with a household income of below $50,000 per year were defined as “low” income constituting 33% of the sample.

**Table 1:** Sample demographics and environmental, and behavioral risk factors for smoking in the 10th grade (*n* = 442), and odds ratios for epigenetic positivity for smoke exposure in 10–12th grade.

**Demographic characteristics and risk factors (10th grade)**	***N* or Mean (*SD* or Percent)**
Age at intake (years)	15.7 (0.6)
Sex (M)	197 (45)
Race (Non-white) (*n* = 440)	70 (16)
Ethnicity (Hispanic)	69 (16)
Household income (< $50k/year)	145 (33)
Probe: “My parents know where I am and who I am with when I am not at home.” Answer: “sometimes or rarely” (vs. “always or usually”) (*n* = 440)	43 (10)
Probe: “How many of your friends to your parents know?” Answer: “none” or “a few” (vs. “most” or “all”) (*n* = 440)	119 (27)
Probe: “How many of your best friends smoke cigarettes?” Answer: “most” or “all” (vs. “none” or “a few”) (*n* = 439)	12 (3)
Probe: “How many of your best friends smoke marijuana?” Answer: “most” or “all” (vs. “none” or “a few”) (*n* = 439)	37 (8)
Probe: “How many kids at school smoke cigarettes?” Answer: “most” or “all” (vs. “none” or “a few”) (*n* = 438)	90 (21)
Probe: “How many kids at school use marijuana” Answer: “most” or “all” (vs. “none” or “a few”) (*n* = 439)	154 (35)
^†^Probe: “Do you have a girlfriend/boyfriend who smokes cigarettes?” Answer: “yes” (vs. “no”) (*n* = 248)	36 (15)
^†^Probe: “Do you have a girlfriend/boyfriend who uses marijuana?” Answer: “yes” (vs. “no”) (*n* = 244)	62 (25)
Probe: “Do you have a family member who smokes” Answer: “yes” (vs. “no”) (*n* = 442)	120 (27)
Probe: “Would you be willing to smoke a single cigarette?” Answer: “very” or “kind of” willing (vs. “not at all”) (*n* = 438)	27 (6)
Probe: “Would you be willing to smoke a single joint?” Answer: “very” or “kind of” willing (vs. “not at all”) (*n* = 438)	73 (17)
Smoker Prototype Scale score > 11 (73rd percentile) (*n* = 438)	120 (27)
Cannabis User Prototype Scale score> 14 (79th percentile) (*n* = 444)	94 (21)
ADHD Symptoms – “high” (≥6 symptoms of inattention and/or hyperactivity/impulsivity) (*n* = 442)	104 (24)
ODD Symptoms –“high” (≥4 symptoms) (*n* = 434)	62 (14)
CD Symptoms – “high” (≥3 or more symptoms) (*n* = 442)	69 (16)
MDD Symptoms – “high” (PHQ-9 score ≥ 9)	59 (13)

*ADHD, Attention-Deficit/Hyperactivity Disorder; ODD, Oppositional-Defiant Disorder; CD, Conduct Disorder*.

† *Only participants who endorsed having a boyfriend/girlfriend were asked this question*.

Students' responses to interview questions are also provided in Table 1. As shown in the table, 10% of 10th graders reported their parents “rarely” or “never” knew where they were when not at home, and 27% reported their parents knew “only a few” or “none” of their friends. Having a family member who smoked was reported by 27% of subjects. Subjects reported higher rates of cannabis smoking compared to cigarette smoking among their best friends (“most or all.” 8 vs. 3%), boyfriend/girlfriend (25 vs. 15%) and other students at their school (“most or all,” 35 vs. 21%). Similarly, subjects reported greater willingness (“kind of willing” or “very willing” vs. “not at all willing”) to smoke a cannabis joint (17%) than a cigarette (6%).

Subjects were more likely to report favorable perceptions of cannabis smokers than cigarette smokers, with the mean Prototype Score for cannabis users equal to 11.4 (SD 3.7) vs. 9.7 (SD 2.8) for smokers. The difference was highly significant (Wilcoxon signed-rank test W = 70,787, *p* = 1.139e-12). Due to the different distributions of each Prototype Score, slightly different cutoffs were used to dichotomize subjects into “high” and “low” risk groups, with the cutoff for cannabis equal to 14 (79%ile) and cigarettes equal to 12 (73%ile).

### Biomarker and Self-Report Relations

At the 10th grade timepoint, 393 of 437 (90.0%) subjects were negative for all three biomarkers, a figure which declined to 323 (85.7%) in 11th grade and 277 (75.9%) by 12th grade. At each timepoint there were subjects positive for one, two, or all three of the biomarkers, with no perfectly overlapping groups. Each timepoint included subjects positive for cg05575921 (methylation <80%) that would not have been identified by the serum biomarkers. A total of 24 (5.4%), 21 (6%), and 25 (7%) demonstrated epigenetic positivity for smoke exposure at the 10, 11, and 12th grade timepoints, respectively.

Self-report relationships revealed interesting patterns. In 10th grade, all e-cigarette users were also cigarette users, whereas in 11 and 12th grade the two groups had significantly less overlap. In terms of self-reported cannabis use, roughly one third of users at both the 10th grade and 12th grade timepoints reported also using either cigarettes or e-cigarettes. Relationships between positive self-report of the use of cigarettes, e-cigarettes, and cannabis at each timepoint are available in the form of Venn diagrams as Supplementary Figure 1, which also depicts relationships between positivity for each biomarker at all three timepoints. Supplementary Table 1 additionally provides the number of subjects positive for cotinine, THC, or either one across the three timepoints in each possible combination (e.g., how many subjects were positive for cotinine in 12th grade but not 10th or 11th).

**Figure 1:**
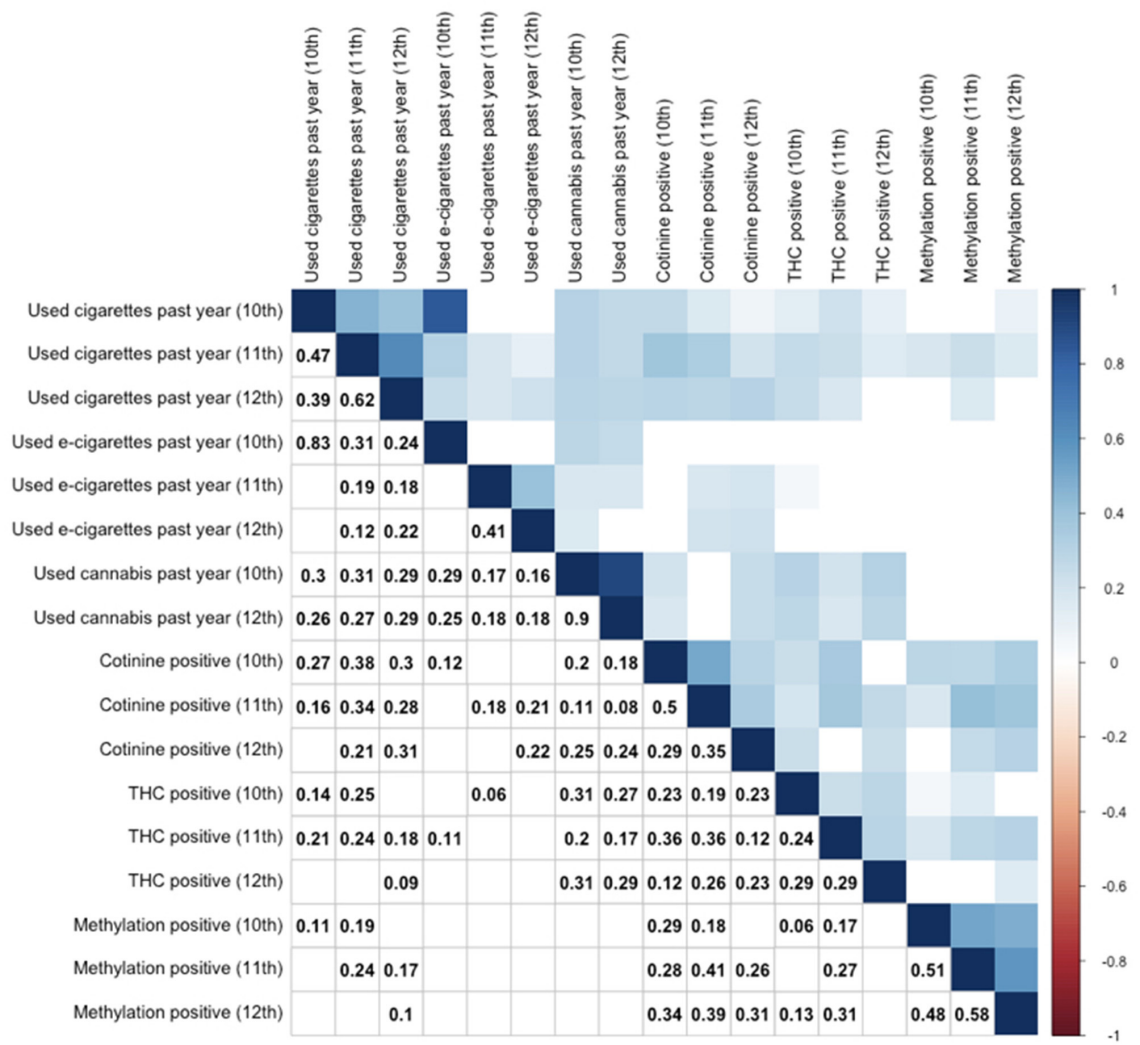
Correlation table for substance use by self-report and biomarker positivity across 10–12th grades. Use of cigarettes, e-cigarettes, and cannabis are by self-report. Other variables reflect objective biomarkers. Cotinine positive indicates serum cotinine (>3 ng/mL). THC positive indicates serum THC (>0.5 ng/mL). Only significant (*p* < 0.05) correlations are provided numerically (lower triangle) or by color (upper triangle), while non-significant correlations are left as blank squares. Correlation values represent Spearman rank correlation coefficients.

A correlation table depicting relationships between self-report measures and biomarkers across all three timepoints is provided as Figure 1. As shown, there were significant relationships between self-reported cigarette use and cotinine cross-sectionally and across timepoints. Relationships between cotinine and self-reported e-cigarette were weaker but still significant cross-sectionally. Interestingly, cotinine positivity was also significantly related to self-reported cannabis use, particularly at the 12th grade timepoint. Epigenetic positivity was significantly related to self-reported cigarette use cross-sectionally and robustly related to cotinine positivity across timepoints, and to a lesser degree, THC positivity. Interestingly, it was unrelated to self-reported past year e-cigarette use and cannabis use at any timepoint. Finally, and unsurprisingly, biomarker positivity at one timepoint was generally associated with positivity for the same biomarker at other timepoints.

To address the issue of subject dropout between 10 and 12th grade influencing the above analyses, we tested whether subjects who completed their 10th grade but not 12th grade visit had differing rates of biomarker positivity. Chi-square analysis of each biomarker vs. dropout by 12th grade revealed no significant relationships (all *p* > 0.05).

Given the modest associations between positivity serum biomarkers (cotinine and THC) and cg05575921 methylation at the chosen cutoffs, these relationships were examined in a linear fashion using simple regression models. As shown in Supplementary Figure 2, linear relationships between cg05575921 methylation and serum cotinine (a) and serum THC (b) were stronger than for the binary relationships (all *p* < 0.001). Linear relationships between cg05575921 methylation and cotinine were stronger (*R* = −0.4 to −0.55) than those between cg05575921 methylation and THC (−0.21 to −0.37) at each timepoint, and consistent with our prior findings (69).

### Effect of Tobacco and Cannabis Co-use on Methylation Levels

**Figure 2:**
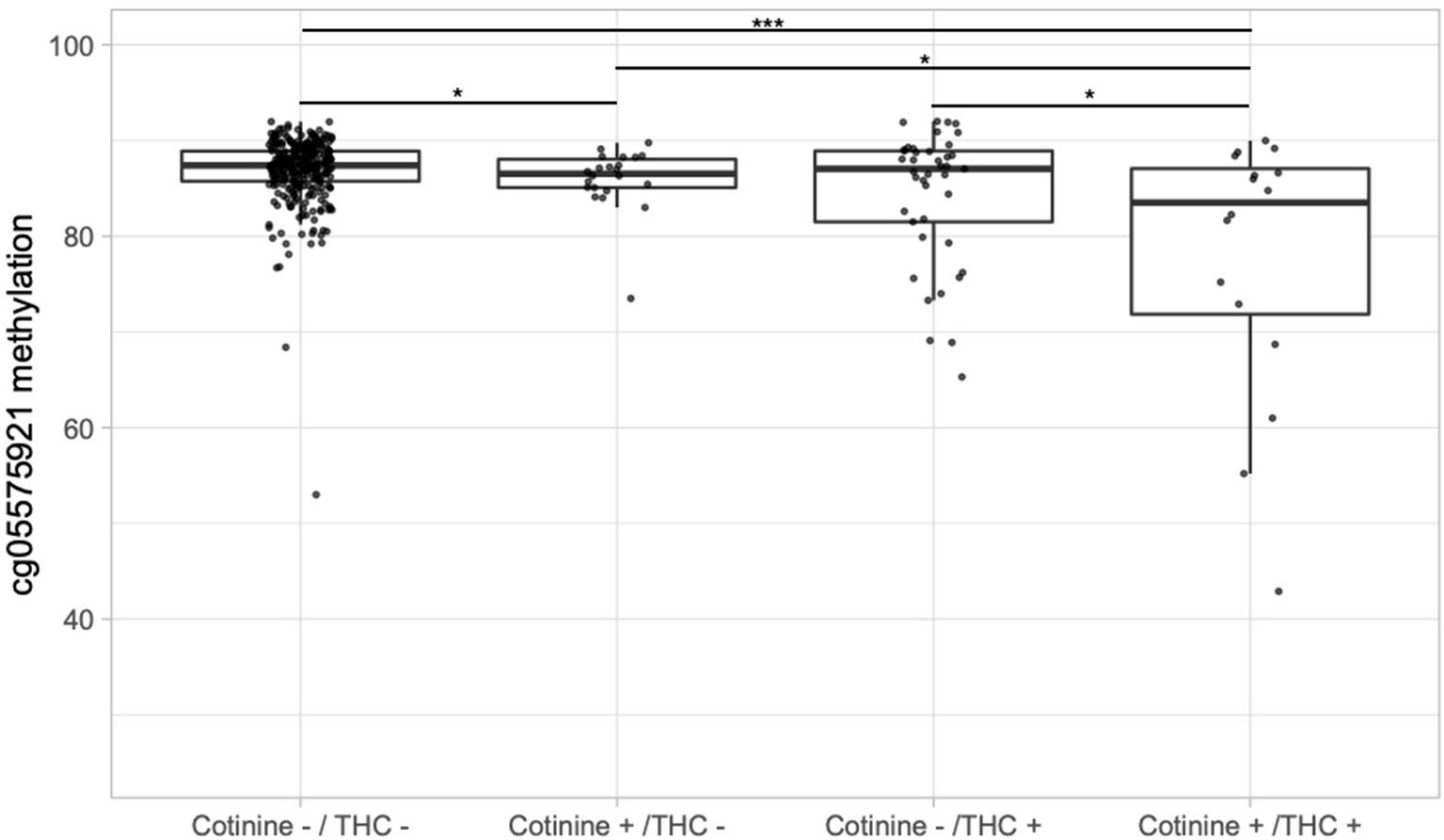
Boxplots of DNA methylation at cg05575921 vs. serum cotinine and/or THC positivity in the 12th grade. “Cotinine +” indicates > 3 ng/mL and “THC +” indicates > 0.5 ng/mL. Significance codes: *p ≤ 0.05; **p ≤ 0.01; ***p ≤ 0.001 (Tukey's Honest Significant Difference).

Next, we examined whether adolescents with serum evidence of tobacco and cannabis co-use had greater epigenetic changes. Figure 2 depicts boxplots of DNA methylation at cg05575921 for the 12th grade timepoint stratified by cotinine positivity vs. negativity and THC positivity vs. negativity. Those positive for either THC (85.9%) or cotinine (mean 84.4%) had lower average methylation than those negative for both (mean 86.8%), and the difference was significant between those positive for cotinine only and those negative for both (*p* < 0.05). Those positive for both THC and cotinine had the lowest average methylation (mean 77.5%), and their average methylation was significantly different from each of the other groups (all *p* < 0.05), and most significantly different from those negative for both (*p* < 0.001).

To address the possibility that lower methylation cg05575921 methylation levels in tobacco and cannabis dual users was simply due to greater tobacco smoke exposure, we examined whether linear regression models predicting methylation levels would show a significant effect of serum THC after adjustment for serum cotinine. At the 10th grade timepoint, THC was not a significant predictor of methylation (*p* = 0.29) with the inclusion of cotinine as an additional predictor. However, at both the 11th grade and 12th grade timepoints, THC was a highly significant predictor (both *p* < 0.001). Further, the addition of THC to cotinine as a linear predictor of cg05575921 methylation significantly improved model fit at both the 11th grade [*F*_(1,378)_ = 46.58, *p* < 0.001] and 12th grade [*F*_(1,366)_ = 29.42, *p* < 0.001] timepoints compared to cotinine alone.

### Risk Factors Predicting Biomarker Positivity

Subjects provided information on a variety of risk factors known to be associated with adolescent smoking, including their family environment, peer and school environment, personal willingness, and smoker Prototypes (tobacco and cannabis) (58, 59, 70), and internalizing and externalizing psychopathology. These risk factors were then used as predictors of positivity for epigenetic positivity and serum cotinine and THC positivity at the 10, 11, and 12th grade timepoints.

**Figure 3:**
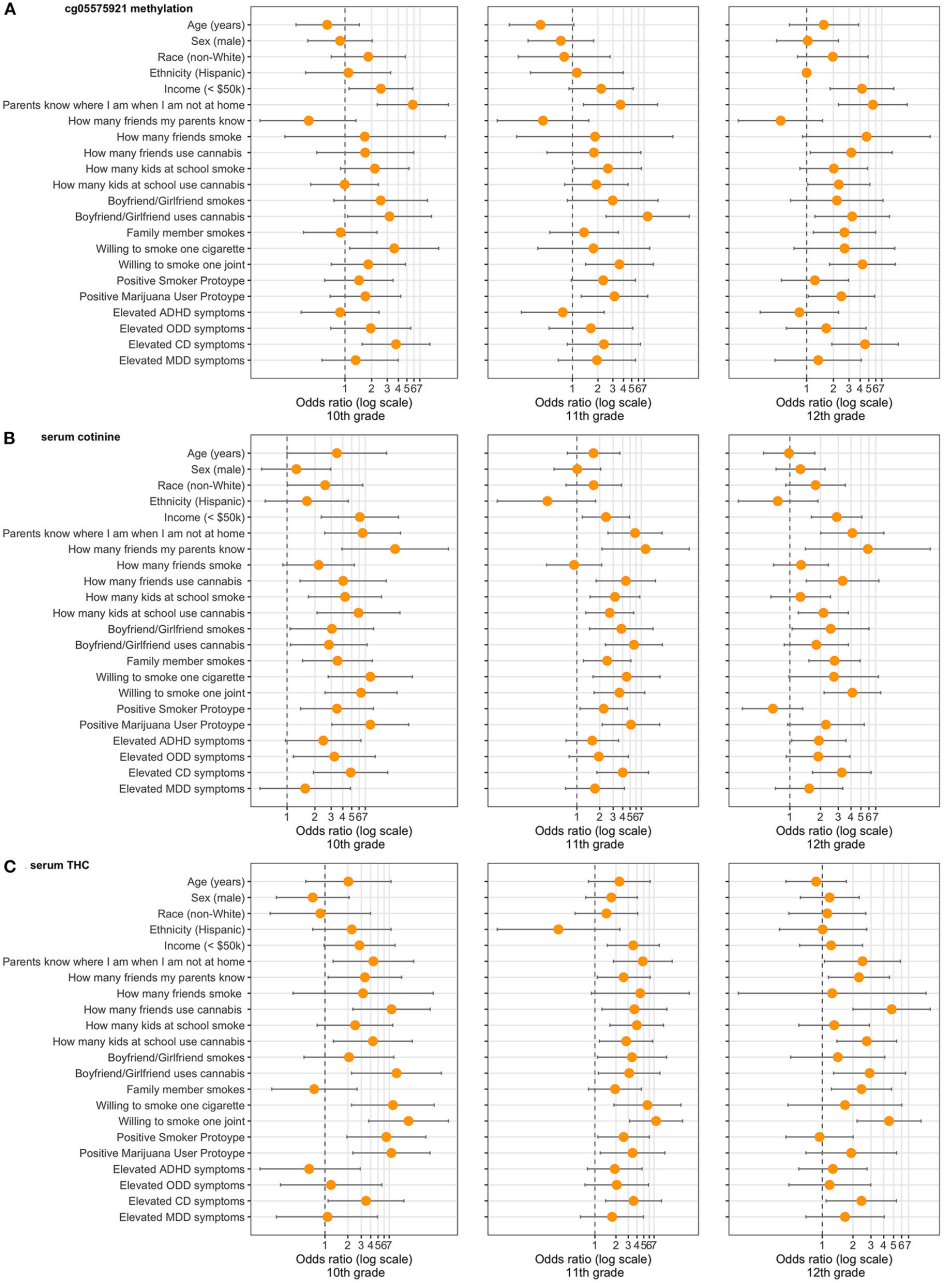
Demographic, environmental, and behavioral risk factors ascertained in 10th grade and Odds Ratios for **(A)** epigenetic positivity (cg05575921 methylation), **(B)** cotinine positivity (>3 ng/mL), and **(C)** THC positivity (>0.5 ng/mL) positivity in 10–12th grade (*n* = 442). Only participants who endorsed having a boyfriend/girlfriend were asked questions regarding the significant other using cigarettes or cannabis. “Parents know where I am when not at home” refers to “sometimes or rarely.” “How many friends my parents know” refers to “none or a few.” “How many friends smoke” and “How many friends use cannabis” refer to “most or all.” “How many kids at school smoke” and “How many kids at school use cannabis” refers to “most or all.” “Willing to smoke one cigarette” and “Willing to smoke one joint” refer to “very or kind of.” “Positive Smoker Prototype” refers to 73rd percentile and above. “Positive Marijuana User Prototype” refers to 79th percentile and above. “Elevated ADHD symptoms” refers to 6 or more inattentive and/or hyperactive/impulsive. “Elevated ODD symptoms” refers to 4 or more. “Elevated CD symptoms” refers to 3 or more. “Elevated MDD symptoms” refers to 9 or more on the PHQ-9. All Odds Ratios computed by unconditional maximum likelihood (Wald) tests.

Figure 3 depicts the calculated Odds Ratio (OR) of positivity for our epigenetic biomarker and our serum biomarkers, cotinine and THC, across the three timepoints, also available as Supplementary Tables 2–4. Note that for the 10th grade timepoint, these ORs represent cross-sectional rather than prospective associations. Broadly speaking, the number of risk factors for epigenetic positivity increased from 10th grade to 12th grade, while the opposite was true for cotinine and THC positivity. For example, in the 10th grade, low income was associated with a significantly increased OR (2.54) of epigenetic positivity, while sex and race were not associated. Other risk factors significantly associated with epigenetic positivity in 10th grade included low parental supervision, a significant other using cannabis, and willingness to smoke a cigarette. By 12th grade, best friends using cannabis, use of cannabis by peers, willingness to smoke a joint, and subjects' perception of cannabis users (Prototype Score) all became significant predictors, as did family member cigarette smoking.

For cotinine, nearly every risk factor was significantly associated with positivity at the 10th grade timepoint. By 12th grade, the strongest risk factors for cotinine positivity included low parental supervision, how many friends used cannabis, and willingness to smoke one joint. For THC positivity, the most associated risk factor in 10th grade was willingness to smoke one joint. By 12th grade, the number of friends using cannabis became the most significant risk factor.

Across timepoints and biomarkers, parental supervision was the risk factor most consistently associated with positivity, being a significant predictor in each analysis. Having a significant other who used cannabis, willingness to smoke one joint, and high levels of CD symptoms were significant predictors in each analysis but one.

## Discussion

In this study we examined relationships between various risk factors for tobacco and cannabis smoking and an emerging epigenetic biomarker for smoke exposure, DNA methylation of the genomic CpG cg05575921. Our Eastern Iowa adolescents' responses reflect broader changes seen over the past two decades in that many more reported having friends and significant others who used cannabis compared to smoking cigarettes. Similarly, adolescents perceived cannabis users more positively than cigarette smokers, and were more willing to smoke a joint than a cigarette. Interestingly, these patterns were not reflected in indicators of nicotine (serum cotinine) vs. smoke exposure (cg05575921 methylation) across the 10th grade through 12th grades. The finding that more subjects were positive for serum cotinine than serum THC at each timepoint could be due to e-cigarette use. Examination of our epigenetic biomarker for smoke exposure showed significant overlap with serum biomarkers but also revealed some subjects at each timepoint for whom the smoke exposure would not have been otherwise detected.

Relationships between risk factors and positivity for our biomarkers showed substantial overlap at each timepoint, consistent with expectations that many risk factors for substance use are non-specific. We also observed an increasing proportion of high schoolers demonstrating epigenetic positivity for smoke exposure, defined as cg05575921 methylation <80%, as they progressed from 10 to 12th grade, consistent with decades of research indicating that late adolescence is a period of peak risk for the initiation of smoking. By 12th grade, this resulted in a doubling of risk factors being significantly associated with epigenetic positivity for smoke exposure, whereas an opposite trend was seen for serum cotinine and THC positivity, with fewer risk factors remaining significantly associated in 12th grade as compared to 10 and 11th grade. These contrasting patterns may reflect the fact that we chose fairly low cutoffs for positivity for our serum biomarkers in order to maximize sensitivity. Over time we would thus expect to see decreased ORs for some risk factors as more adolescents in various “low risk” groups begin using. In contrast, because epigenetic positivity requires cumulative exposure to smoke for methylation at cg05575921 to decrease from a population average of around 86% to below 80%, our finding that more risk factors became significant over time is expected.

In terms of specific risk factors analyzed, it is not surprising that lower parental supervision was robustly associated with an increased odds of biomarker positivity. Best friends' and significant others' use of cannabis were significant risk factor for positivity for multiple biomarkers at multiple timepoints. Whereas, low income was strongly associated with increased risk, race and ethnicity were not associated with a greater or lower risk, though this negative finding may reflect the relatively low number of non-white and Hispanic subjects in our sample. Similarly, because only 3% of subjects reported they had best friends who smoked cigarettes in 10th grade, our study may not have been adequately powered to examine this risk factor. As expected, both willingness to smoke cigarettes and cannabis as well as prototypes of smokers of each substance were associated with positivity at one or more timepoints for each biomarker (59). Lastly, CD symptoms were significantly associated with positivity for each biomarker at each timepoint with the exception of epigenetic positivity in 11th grade. In contrast, although ADHD and ODD symptoms are known risk factors for smoking, these associations were generally not significant in our sample, nor were depressive symptoms.

Our results also provide evidence that co-users of tobacco and cannabis on average have greater cumulative exposure to smoke and its toxic components (44), including PAHs (71). By 12th grade, those positive for both cotinine and THC showed a significantly lower average methylation at cg05575921 than those positive for THC alone (*p* < 0.001), cotinine alone (*p* < 0.001), or negative for both (*p* < 0.001). This is consistent with two recent studies reporting highly significant demethylation of cg05575921 in cannabis and tobacco co-users (72, 73). Interestingly, Osborne and colleagues (73) reported no CpGs significantly associated with exclusive cannabis use, suggesting that tobacco use may dominate or mask signatures specific to cannabis and/or THC. To address this possibility, the recent study of Markunas et al. (74) controlled for tobacco exposure in their epigenome-wide association study of lifetime cannabis use, finding modest evidence of association with the CpG cg15973234 in the gene *CEMIP*.

An alternative interpretation of our findings is that co-users of tobacco and cannabis may simply be exposed to more tobacco smoke than tobacco smokers who do not use cannabis. Although limitations in our self-report data do not allow us to examine this possibility directly, linear regression models controlling for serum cotinine did show a significant independent effect of serum THC on cg05575921 methylation at the 11 and 12th grade timepoints, suggesting greater tobacco smoke exposure is not solely responsible for lower methylation in the dual-use group. In addition, *post-hoc* analysis of serum cotinine and THC values in the 12th grade suggest greater change in cg05575921 methylation in dual users may be due to heavier cannabis use. Mean serum cotinine was slightly higher in the cotinine positive, THC negative group (53.1 ng/mL) than in the group positive for both substances (41.9 ng/mL), whereas mean serum THC levels were higher in the dual use group (4.9 ng/mL) than in the group only positive for THC (2.7 ng/mL). However, the latter difference was only significant at the trend level (*p* < 0.10).

E-cigarette use in our study was not significantly correlated with demethylation of cg05575921, despite the fact that e-cigarette use predisposes to future combusted tobacco use (20). This is likely because e-cigarettes are not thought to expose users to sufficient levels of PAHs (75) to induce demethylation of cg05575921. E-cigarette use may also explain the weaker correlations between cotinine positivity and epigenetic positivity compared to self-reported cigarette smoking and epigenetic positivity, as the cotinine positive group likely contains both cigarette users and e-cigarette users.

In considering the results above, several limitations or alternate explanations are worth mentioning. First, secondhand smoke exposure may explain the association between some risk factors and epigenetic positivity, particularly the presence of smokers in the home, although this effect is likely modest (76). A second limitation, subject dropout, is a potential concern as this could bias observed relationships between risk factors and biomarker positivity. It would be expected that dropouts would be those more likely to be engaging in risk behaviors in general. However, analysis of subjects who completed the 10th grade but not 12th grade visits did not reveal significant differences between groups for any biomarker in 10th grade. Third, although we selected adolescents at higher risk than the general population due to having a friend or family member who smokes cigarettes, our sample had a limited proportion of socioeconomically disadvantaged and ethnic/racial minority subjects, limiting generalizability to these groups. Our small sample size may also have limited our ability to detect significant associations between some risk factors and biomarker positivity, particularly at the 12th grade timepoint.

The most significant limitation of the epigenetic technology discussed above may be its insensitivity to pure e-cigarette use. The large proportion of subjects were e-cigarette users in 12th grade (54/364) may also explain our finding that the correlations between cg05575921 methylation and cotinine positivity at each timepoint, though significant, were modest (0.29 – 0.41), with linear relationships being somewhat stronger. This suggests the need to develop more specific biomarkers for non-combusted nicotine use, and that cg05575921 may be best thought of as an additional screening tool rather than replacement for conventional biomarkers and other methods of detecting substance use in adolescents. The high rate of e-cigarette use also suggests that public health campaigns designed to shift adolescents' perception of “typical” e-cigarette users (Prototypes) toward the negative may be helpful, similar to past effects observed with cigarette smokers (77).

Ethical concerns in the use of an epigenetic biomarker to study smoking in adolescent populations are an important consideration. While these concerns should be balanced against the public health impact of smoking, the largest preventable cause of mortality in the US, it is important to note that laboratory testing, whether conventional or epigenetic, is generally not supported as a standalone screening or assessment for substance use (78), and that children and adolescents are inherently more vulnerable than adults, placing greater burden on the clinician in weighing the costs and benefits of such testing.

In summary, the epigenetic biomarker cg05575921 can provide a window into changing patterns of nicotine, tobacco, and cannabis use in adolescence. Our analyses suggest that in addition to being a highly sensitive biomarker for tobacco smoke, cg05575921 methylation may also be a novel tool for detecting cannabis smoking in adolescents, and further study of its utility in larger samples of pure cannabis users is warranted. Going forward, cg05575921 may have a role in as a clinical screening tool, complementing self-report and other methods of assessing substance use in adolescents. At the same time, because the biomarker is insensitive to pure e-cigarette use, other screening methods, including high quality interview-based measures (79), will continue to be essential clinical tools.

## Presentation Information

This study was presented as an abstract at the American Academy of Child and Adolescent Psychiatry's 67th Virtual Annual Meeting, October 12–24th, 2020.

## Data Availability Statement

The raw data supporting the conclusions of this article will be made available by the authors, without undue reservation.

## Ethics Statement

The studies involving human participants were reviewed and approved by University of Iowa's Institutional Review Board (IRB ID # 201409705). Written informed consent to participate in this study was provided by the participants' legal guardian/next of kin.

## Author Contributions

RP and MG obtained funding for this study and developed key concepts. AA performed the initial analyses and wrote the manuscript. RP, MG, and FG edited the manuscript extensively. All authors contributed to the article and approved the submitted version.

## Conflict of Interest

RP is the Chief Executive Officer of Behavioral Diagnostics and inventor on a number of granted and pending patent applications with respect to both alcohol and tobacco consumption related to the material discussed herein. The use of cg05575921 status to determine smoking status is protected by US Patents 8,637,652 and 9,273,358. The remaining authors declare that the research was conducted in the absence of any commercial or financial relationships that could be construed as a potential conflict of interest.

## Publisher's Note

All claims expressed in this article are solely those of the authors and do not necessarily represent those of their affiliated organizations, or those of the publisher, the editors and the reviewers. Any product that may be evaluated in this article, or claim that may be made by its manufacturer, is not guaranteed or endorsed by the publisher.

## Supplementary Material

Supplemental Figure 1 Venn diagrams of **(A)** positivity for serum cotinine (>3 ng/mL), serum THC (>0.5 ng/mL), and cg05575921 methylation (<80%) and **(B)** self-reported use of cannabis, cigarettes, and e-cigarettes in the past year, 10–12th grades. Self-reported cannabis use was not available for analysis at the 11th grade timepoint.

Supplemental Figure 2 Linear fits of cg05575921 methylation vs. serum cotinine and THC at the **(A)** 10th, **(B)** 11th, and **(C)** 12th grade timepoints.

Supplementary Table 1 Positivity for cotinine, THC, or either at 10, 11, and 12th grades.

Supplementary Table 2 Environmental, and behavioral risk factors for smoking in the 10th grade (*n* = 442), and odds ratios for epigenetic positivity for smoke exposure in 10–12th grade.

Supplementary Table 3 Demographic, environmental, and behavioral risk factors ascertained in 10th grade and odds ratios for cotinine positivity in 10–12th grade (*n* = 442).

Supplementary Table 4 Demographic, environmental, and behavioral risk factors ascertained in 10th grade and odds ratios for THC positivity in 10–12th grade (*n* = 442).
