# Supplementary figures and images for: An Examination of Risk Factors for Tobacco and Cannabis Smoke Exposure in Adolescents Using an Epigenetic Biomarker
Source: Front Psychiatry. 2021 Aug 24;12:688384. doi: 10.3389/fpsyt.2021.688384 (PMC8421639; doi:10.3389/fpsyt.2021.688384)

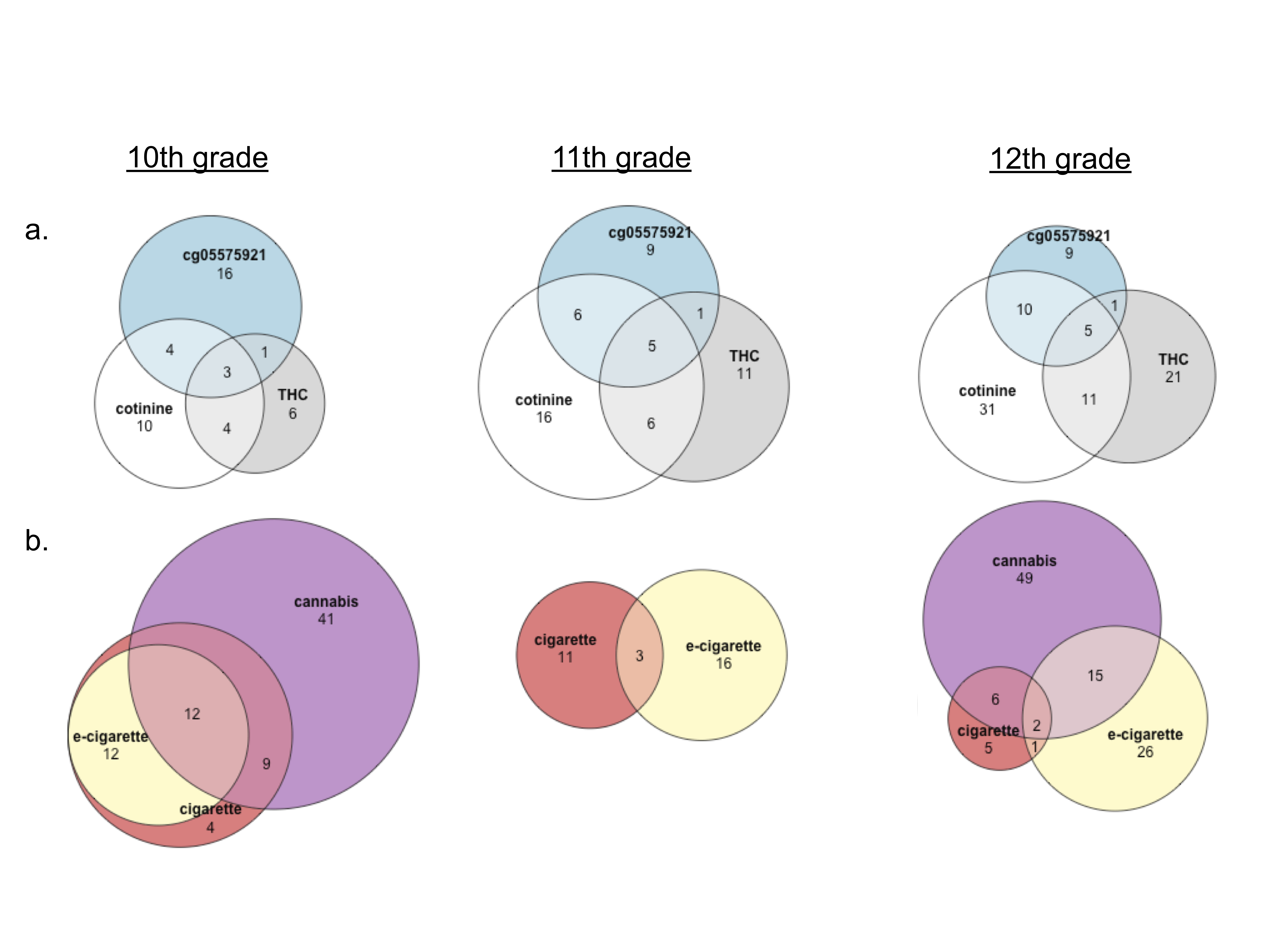

Supplement: Supplemental Figure 1 — Venn diagrams of (A) positivity for serum cotinine (>3 ng/mL), serum THC (>0.5 ng/mL), and cg05575921 methylation (<80%) and (B) self-reported use of cannabis, cigarettes, and e-cigarettes in the past year, 10–12th grades. Self-reported cannabis use was not available for analysis at the 11th grade timepoint. [file Image_1.JPEG]

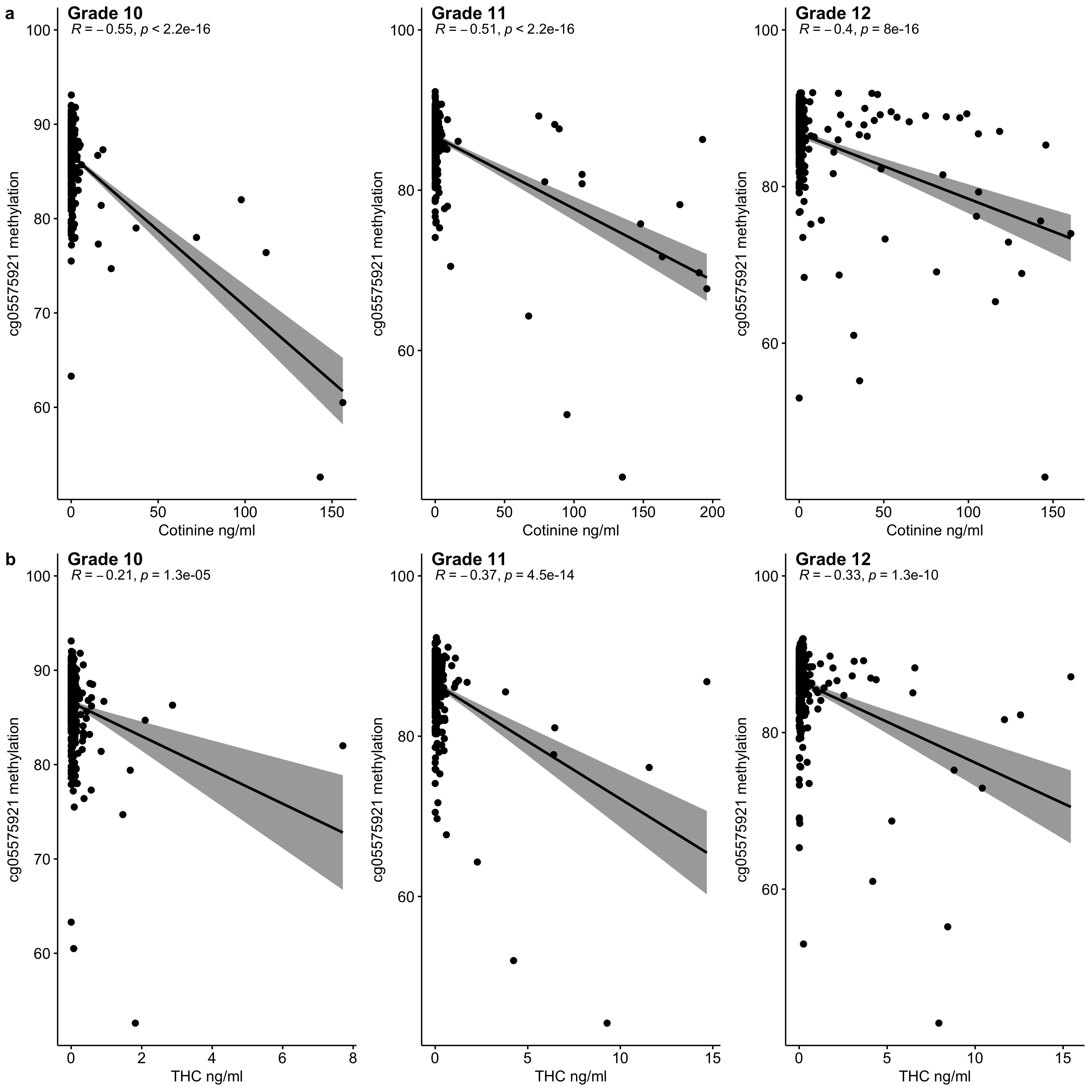

Supplement: Supplemental Figure 2 — Linear fits of cg05575921 methylation vs. serum cotinine and THC at the (A) 10th, (B) 11th, and (C) 12th grade timepoints. [file Image_2.JPEG]
