# Supplementary material for: An Examination of Risk Factors for Tobacco and Cannabis Smoke Exposure in Adolescents Using an Epigenetic Biomarker
Source: Front Psychiatry. 2021 Aug 24;12:688384. doi: 10.3389/fpsyt.2021.688384 (PMC8421639; doi:10.3389/fpsyt.2021.688384)
Supplement: Supplementary Table 2 — Environmental, and behavioral risk factors for smoking in the 10th grade (n = 442), and odds ratios for epigenetic positivity for smoke exposure in 10–12th grade. [file Table_2.docx]

Supplemental Table 2. Environmental, and Behavioral Risk Factors for Smoking in the 10th grade (n = 442), and Odds Ratios for epigenetic positivity for smoke exposure in 10^th^-12^th^ grade.

| Risk Factor (10^th^ grade) | Epigenetic positivity (10^th^ grade) | Epigenetic positivity (11^th^ grade) | Epigenetic positivity (11^th^ grade) |
| --- | --- | --- | --- |
| Age at intake (years) | 0.63 (0.28, 1.45) | 0.42 (0.18, 1.04) | 1.56 (0.64, 3.85) |
| Sex (M) | 0.88 (0.38, 2.03) | 0.73 (0.30, 1.78) | 1.03 (0.46, 2.28) |
| Race (Non-white) (n = 440) | 1.83 (0.70, 4.80) | 0.80 (0.23, 2.78) | 1.97 (0.79, 4.92) |
| Ethnicity (Hispanic) | 1.09 (0.36, 3.28) | 1.13 (0.32, 3.98) | - |
| Household income (<$50k/year) | **2.54 (1.11, 5.83)** | 2.18 (0.91, 5.21) | **4.19 (1.84, 9.57)** |
| Probe: “My parents know where I am and who I am with when I am not at home.”  Answer: “sometimes or rarely” (versus “always or usually”) (n = 440) | **5.82 (2.31, 14.68)** | **3.69 (1.35, 10.08)** | **5.56 (2.28, 13.54)** |
| Probe: “How many of your friends to your parents know?”  Answer: “none” or “a few” (versus “most” or “all”) (n = 440) | 0.39 (0.11, 1.33) | 0.45 (0.13, 1.56) | 0.51 (0.17, 1.51) |
| Probe: “How many of your best friends smoke cigarettes?”  Answer: “most” or “all” (versus “none” or “a few”) (n = 439) | 1.67 (0.21, 13.55) | 1.85 (0.22, 15.27) | 4.71 (0.90, 24.59) |
| Probe: “How many of your best friends smoke marijuana?”  Answer: “most” or “all” (versus “none” or “a few”) (n = 439) | 1.69 (0.48, 5.96) | 1.79 (0.50, 6.41) | **3.18 (1.10, 9.19)** |
| Probe: “How many kids at school smoke cigarettes?”  Answer: “most” or “all” (versus “none” or “a few”) (n = 438) | 2.17 (0.89, 5.28) | **2.62 (1.05, 6.50)** | 2.02 (0.84, 4.86) |
| Probe: “How many kids at school use marijuana”  Answer: “most” or “all” (versus “none” or “a few”) (n = 439) | 0.99 (0.41, 2.38) | 1.91 (0.81, 4.53) | **2.30 (1.03, 5.14)** |
| ^†^Probe: “Do you have a girlfriend/boyfriend who smokes cigarettes?”  Answer: “yes” (versus “no”) (n = 248) | 2.53 (0.75, 8.54) | 2.98 (0.87, 10.20) | 2.19 (0.66, 7.24) |
| ^†^Probe: “Do you have a girlfriend/boyfriend who uses marijuana?”  Answer: “yes” (versus “no”) (n = 244) | **3.18 (1.07, 9.47)** | **7.69 (2.49, 23.81)** | **3.25 (1.24, 8.56)** |
| Probe: “Do you have a family member who smokes”  Answer: “yes” (versus “no”) (n = 442) | 0.89 (0.34, 2.30) | 1.37 (0.54, 3.48) | **2.66 (1.19, 5.98)** |
| Probe: “Would you be willing to smoke a single cigarette?”  Answer: “very” or “kind of” willing (versus “not at all”) ( n = 438) | **3.59 (1.13, 11.42)** | 1.77 (0.39, 8.15) | 2.67 (0.72, 9.82) |
| Probe: “Would you be willing to smoke a single joint?”  Answer: “very” or “kind of” willing (versus “not at all”) ( n = 438) | 1.83 (0.70, 4.82) | **3.58 (1.43, 8.98)** | **4.25 (1.81, 9.95)** |
| Smoker Prototype Scale score > 11 (73^rd^ percentile) (n = 438) | 1.44 (0.59, 3.49) | **2.31 (0.97, 5.53)** | 1.24 (0.52, 2.96) |
| Cannabis User Prototype Scale score> 14 (79^th^ percentile) (n = 444) | 1.70 (0.68, 4.26) | **3.13 (1.27, 7.73)** | **2.46 (1.04, 5.83)** |
| ADHD Symptoms – “high” (>= 6 symptoms of inattention and/or hyperactivity/impulsivity) (n = 442) | 0.88 (0.32, 2.43) | 0.77 (0.25, 2.37) | 0.83 (0.30, 2.29) |
| ODD Symptoms –“high” ( >= 4 symptoms) (n = 434) | 1.95 (0.69, 5.52) | 1.65 (0.53, 5.16) | 1.66 (0.59, 4.66) |
| CD Symptoms – “high” (>= 3 or more symptoms) (n = 442) | **3.76 (1.56, 9.08)** | 2.35 (0.87, 6.33) | **4.54 (1.92, 10.75)** |
| MDD Symptoms – “high” (PHQ-9 score >= 9) | 1.32 (0.55, 3.99) | 1.95 (0.68, 5.55) | 1.35 (0.44, 4.13) |

Epigenetic positivity indicates cg05575921 methylation < 80%. Bolded entries indicate the 95% two-sided Confidence Interval does not overlap 1. ADHD Refers to Attention-Deficit/Hyperactivity Disorder. ODD refers to Oppositional-Defiant Disorder. CD refers to Conduct Disorder. MDD refers to Major Depressive Disorder. Of note, because 0% of subjects identifying as Hispanic demonstrated epigenetic positivity at the 12th grade timepoint, an OR for this risk factor could not be calculated. ^†^Only participants who endorsed having a boyfriend/girlfriend were asked this question.
