# Supplementary material for: An Examination of Risk Factors for Tobacco and Cannabis Smoke Exposure in Adolescents Using an Epigenetic Biomarker
Source: Front Psychiatry. 2021 Aug 24;12:688384. doi: 10.3389/fpsyt.2021.688384 (PMC8421639; doi:10.3389/fpsyt.2021.688384)
Supplement: Supplementary Table 3 — Demographic, environmental, and behavioral risk factors ascertained in 10th grade and odds ratios for cotinine positivity in 10–12th grade (n = 442). [file Table_3.docx]

Supplemental Table 3. Demographic, environmental, and behavioral risk factors ascertained in 10^th^ grade and Odds Ratios for Cotinine positivity in 10^th^ – 12^th^ grade (n = 442).

| Risk Factor (10^th^ grade) | Cotinine positivity (10^th^ grade) | Cotinine positivity (11^th^ grade) | Cotinine positivity (12^th^ grade) |
| --- | --- | --- | --- |
| Age at intake (years) | 3.45 (1.00, 11.91) | 1.65 (0.75, 3.67) | 0.98 (0.55, 1.76) |
| Sex (M) | 1.26 (0.53, 2.97) | 1.01 (0.50, 2.06) | 1.27 (0.73, 2.22) |
| Race (Non-white) (n = 440) | **2.58 (1.01, 6.58)** | 1.66 (0.72, 3.86) | 1.79 (0.91, 3.52) |
| Ethnicity (Hispanic) | 1.64 (0.58, 4.59) | 0.41 (0.09, 1.76) | 0.76 (0.31, 1.89) |
| Household income (<$50k/year) | **6.11 (2.34, 15.98)** | **2.41 (1.18, 4.93)** | **2.89 (1.63, 5.12)** |
| Probe: “My parents know where I am and who I am with when I am not at home.”  Answer: “sometimes or rarely” (versus “always or usually”) (n = 440) | **6.55 (2.55, 16.84)** | **5.80 (2.56, 13.18)** | **4.12 (2.01, 8.42)** |
| Probe: “How many of your friends to your parents know?”  Answer: “none” or “a few” (versus “most” or “all”) (n = 440) | **14.71 (3.91, 55.42**) | **7.98 (2.13, 29.88)** | **5.85 (1.42, 24.10)** |
| Probe: “How many of your best friends smoke cigarettes?”  Answer: “most” or “all” (versus “none” or “a few”) (n = 439) | 2.19 (0.90, 5.33) | 0.92 (0.40, 2.12) | 1.29 (0.69, 2.39) |
| Probe: “How many of your best friends smoke marijuana?”  Answer: “most” or “all” (versus “none” or “a few”) (n = 439) | **4.03 (1.38, 11.80)** | **4.39 (1.79, 10.76)** | **3.30 (1.45, 7.52)** |
| Probe: “How many kids at school smoke cigarettes?”  Answer: “most” or “all” (versus “none” or “a few”) (n = 438) | **4.23 (1.70, 10.49)** | **3.16 (1.49, 6.71)** | 1.27 (0.65, 2.51) |
| Probe: “How many kids at school use marijuana”  Answer: “most” or “all” (versus “none” or “a few”) (n = 439) | **5.91 (2.11, 16.60)** | **2.70 (1.31, 5.58)** | **2.14 (1.21, 3.76)** |
| ^†^Probe: “Do you have a girlfriend/boyfriend who smokes cigarettes?”  Answer: “yes” (versus “no”) (n = 248) | **3.05 (1.08, 8.62)** | **3.84 (1.47, 10.01)** | **2.52 (1.05, 6.01)** |
| ^†^Probe: “Do you have a girlfriend/boyfriend who uses marijuana?”  Answer: “yes” (versus “no”) (n = 244) | **2.83 (1.09, 7.33)** | **5.61 (2.37, 13.27)** | 1.82 (0.88, 3.77) |
| Probe: “Do you have a family member who smokes”  Answer: “yes” (versus “no”) (n = 442) | **3.51 (1.47, 8.36)** | **2.50 (1.22, 5.13)** | **2.75 (1.54, 4.91)** |
| Probe: “Would you be willing to smoke a single cigarette?”  Answer: “very” or “kind of” willing (versus “not at all”) ( n = 438) | **7.94 (2.78, 22.64)** | **4.49 (1.63, 12.37)** | 2.71 (0.98, 7.44) |
| Probe: “Would you be willing to smoke a single joint?”  Answer: “very” or “kind of” willing (versus “not at all”) ( n = 438) | **6.30 (2.57, 15.46)** | **3.62 (1.68, 7.80)** | **4.12 (2.17, 7.84)** |
| Smoker Prototype Scale score > 11 (73^rd^ percentile) (n = 438) | **3.46 (1.40, 8.58)** | **2.25 (1.10, 4.61)** | 0.68 (0.34, 1.34) |
| Cannabis User Prototype Scale score> 14 (79^th^ percentile) (n = 444) | **7.94 (3.05, 20.65)** | **5.13 (2.15, 12.27)** | 2.26 (0.95, 5.39) |
| ADHD Symptoms – “high” (>= 6 symptoms of inattention and/or hyperactivity/impulsivity) (n = 442) | 2.46 (0.96, 6.29) | 1.59 (0.72, 3.53) | **1.93 (1.04, 3.57)** |
| ODD Symptoms –“high” ( >= 4 symptoms) (n = 434) | **3.24 (1.17, 8.98)** | 1.94 (0.79, 4.77) | 1.90 (0.92, 3.91) |
| CD Symptoms – “high” (>= 3 or more symptoms) (n = 442) | **4.87 (1.93, 12.25)** | **4.00 (1.83, 8.74)** | **3.24 (1.67, 6.30)** |
| MDD Symptoms – “high” (PHQ-9 score >= 9) | 1.57 (0.51, 4.85) | 1.73 (0.71, 4.21) | 1.55 (0.72, 3.32) |

Cotinine positivity indicates > 3 ng/mL. Bolded entries indicate the 95% two-sided Confidence Interval does not overlap 1. ADHD refers to Attention-Deficit/Hyperactivity Disorder. ODD refers to Oppositional-Defiant Disorder. CD refers to Conduct Disorder. MDD referes to Major Depressive Disorder. ^†^Only participants who endorsed having a boyfriend/girlfriend were asked this question.
