# Supplementary material for: An Examination of Risk Factors for Tobacco and Cannabis Smoke Exposure in Adolescents Using an Epigenetic Biomarker
Source: Front Psychiatry. 2021 Aug 24;12:688384. doi: 10.3389/fpsyt.2021.688384 (PMC8421639; doi:10.3389/fpsyt.2021.688384)
Supplement: Supplementary Table 4 — Demographic, environmental, and behavioral risk factors ascertained in 10th grade and odds ratios for THC positivity in 10–12th grade (n = 442). [file Table_4.docx]

Supplemental Table 4. Demographic, environmental, and behavioral risk factors ascertained in 10^th^ grade and Odds Ratios for THC positivity in 10^th^ – 12^th^ grade (n = 442).

| Risk Factor (10^th^ grade) | THC positivity (10^th^ grade) | THC positivity (11^th^ grade) | THC positivity (12^th^ grade) |
| --- | --- | --- | --- |
| Age at intake (years) | 2.03 (0.56, 7.40) | 2.24 (0.81, 6.16) | 0.87 (0.44, 1.72) |
| Sex (M) | 0.69 (0.23, 2.08) | 1.72 (0.74, 4.03) | 1.18 (0.61, 2.30) |
| Race (Non-white) (n = 440) | 0.87 (0.19, 3.97) | 1.46 (0.52, 4.09) | 1.12 (0.47, 2.67) |
| Ethnicity (Hispanic) | 2.25 (0.69, 7.40) | 0.30 (0.04, 2.28) | 1.01 (0.38, 2.73) |
| Household income (<$50k/year) | 2.84 (0.97, 8.35) | **3.53 (1.50, 8.31)** | 1.22 (0.60, 2.48) |
| Probe: “My parents know where I am and who I am with when I am not at home.”  Answer: “sometimes or rarely” (versus “always or usually”) (n = 440) | **4.33 (1.28, 14.71)** | **4.84 (1.84, 12.74)** | **2.48 (1.05, 5.84)** |
| Probe: “How many of your friends to your parents know?”  Answer: “none” or “a few” (versus “most” or “all”) (n = 440) | **3.35 (1.10, 10.19)** | **2.57 (1.08, 6.16)** | **2.29 (1.15, 4.55)** |
| Probe: “How many of your best friends smoke cigarettes?”  Answer: “most” or “all” (versus “none” or “a few”) (n = 439) | 3.17 (0.38, 26.54) | 4.45 (0.89, 22.34) | 1.25 (0.15, 10.42) |
| Probe: “How many of your best friends smoke marijuana?”  Answer: “most” or “all” (versus “none” or “a few”) (n = 439) | **7.50 (2.32, 24.22)** | **3.67 (1.26, 10.72)** | **4.79 (2.00, 11.44)** |
| Probe: “How many kids at school smoke cigarettes?”  Answer: “most” or “all” (versus “none” or “a few”) (n = 438) | 2.49 (0.79, 7.79) | **3.97 (1.64, 9.59)** | 1.31 (0.59, 2.90) |
| Probe: “How many kids at school use marijuana”  Answer: “most” or “all” (versus “none” or “a few”) (n = 439) | **4.27 (1.29, 14.11)** | **2.80 (1.16, 6.72)** | **2.73 (1.39, 5.35)** |
| ^†^Probe: “Do you have a girlfriend/boyfriend who smokes cigarettes?”  Answer: “yes” (versus “no”) (n = 248) | 2.07 (0.53, 8.05) | **3.39 (1.09, 10.58)** | 1.42 (0.49, 4.10) |
| ^†^Probe: “Do you have a girlfriend/boyfriend who uses marijuana?”  Answer: “yes” (versus “no”) (n = 244) | **8.73 (2.24, 34.03)** | **3.09 (1.12, 8.50)** | **2.91 (1.29, 6.54)** |
| Probe: “Do you have a family member who smokes”  Answer: “yes” (versus “no”) (n = 442) | 0.72 (0.20, 2.64) | 1.94 (0.81, 4.62) | **2.42 (1.23, 4.78)** |
| Probe: “Would you be willing to smoke a single cigarette?”  Answer: “very” or “kind of” willing (versus “not at all”) ( n = 438) | **7.83 (2.24, 27.33)** | **5.64 (1.87, 17.00)** | 1.67 (0.46, 6.02) |
| Probe: “Would you be willing to smoke a single joint?”  Answer: “very” or “kind of” willing (versus “not at all”) ( n = 438) | **12.57 (3.76, 42.01)** | **7.49 (3.13, 17.97)** | **4.51 (2.19, 9.30)** |
| Smoker Prototype Scale score > 11 (73^rd^ percentile) (n = 438) | **6.42 (1.94, 21.27)** | **2.57 (1.10, 6.01)** | 0.94 (0.44, 2.01) |
| Cannabis User Prototype Scale score> 14 (79^th^ percentile) (n = 444) | **7.48 (2.32, 24.16)** | **3.45 (1.19, 10.00)** | 1.92 (0.69, 5.35) |
| ADHD Symptoms – “high” (>= 6 symptoms of inattention and/or hyperactivity/impulsivity) (n = 442) | 0.62 (0.14, 2.91) | 1.92 (0.78, 4.73) | 1.27 (0.59, 2.75) |
| ODD Symptoms –“high” ( >= 4 symptoms) (n = 434) | 1.20 (0.26, 5.60) | 2.05 (0.72, 5.85) | 1.18 (0.47, 2.99) |
| CD Symptoms – “high” (>= 3 or more symptoms) (n = 442) | **3.47 (1.10, 10.93)** | **3.57 (1.42, 8.95)** | **2.42 (1.09, 5.33)** |
| Depressive Symptoms – “high” (PHQ-9 score >= 9) | 1.08 (0.23, 4.93) | 1.76 (0.62, 4.95) | 1.67 (0.69, 4.05) |

THC positivity refers to > 0.5 ng/mL. Bolded entries indicate the 95% two-sided Confidence Interval does not overlap 1. ADHD refers to Attention-Deficit/Hyperactivity Disorder. ODD refers to Oppositional-Defiant Disorder. CD refers to Conduct Disorder. MDD refers to Major Depressive Disorder. ^†^Only participants who endorsed having a boyfriend/girlfriend were asked this question.
